# A Prospective Stroke Register in Sierra Leone: Demographics, Stroke Type, Stroke Care and Hospital Outcomes

**DOI:** 10.3389/fneur.2021.712060

**Published:** 2021-09-07

**Authors:** Daniel Youkee, Gibrilla Deen, Edward Barrett, Julia Fox-Rushby, Israel Johnson, Peter Langhorne, Andrew Leather, Iain J. Marshall, Jessica O'Hara, Anthony Rudd, Albert Sama, Christella Scott, Melvina Thompson, Hatem Wafa, Jurate Wall, Yanzhong Wang, Caroline Watkins, Charles Wolfe, Durodami Radcliffe Lisk, Catherine Mary Sackley

**Affiliations:** ^1^School of Population Health and Environmental Sciences, King's College London, London, United Kingdom; ^2^College of Medicine and Allied Health Sciences, University of Sierra Leone, Freetown, Sierra Leone; ^3^National Institute for Health Research, Biomedical Research Centre, Guy and ST Thomas' NHS Foundation Trust and King's College London, London, United Kingdom; ^4^Institute of Cardiovascular and Medical Sciences, University of Glasgow, Glasgow, United Kingdom; ^5^King's Centre for Global Health and Health Partnerships, School of Population Health and Environmental Sciences, King's College London, London, United Kingdom; ^6^NIHR Applied Research Collaboration South London, London, United Kingdom; ^7^Faculty of Health and Care, University of Central Lancashire, Preston, United Kingdom; ^8^Division of Stroke Medicine, University of Nottingham, Nottingham, United Kingdom

**Keywords:** stroke, registry, Sierra Leone, intracerebral haemorrhage, lower middle income country, Sub-Saharan Africa, cerebrovascular accident

## Abstract

**Introduction:** Stroke is the second most common cause of adult death in Africa. This study reports the demographics, stroke types, stroke care and hospital outcomes for stroke in Freetown, Sierra Leone.

**Methods:** A prospective observational register recorded all patients 18 years and over with stroke between May 2019 and April 2020. Stroke was defined according to the WHO criteria. Pearson's chi-squared test was used to examine associations between categorical variables and unpaired *t*-tests for continuous variables. Multivariable logistic regression, to explain in-hospital death, was reported as odds ratios (ORs) and 95% confidence intervals.

**Results:** Three hundred eighty-five strokes were registered, and 315 (81.8%) were first-in-a-lifetime events. Mean age was 59.2 (SD 13.8), and 187 (48.6%) were male. Of the strokes, 327 (84.9%) were confirmed by CT scan. Two hundred thirty-one (60.0%) were ischaemic, 85 (22.1%) intracerebral haemorrhage, 11 (2.9%) subarachnoid haemorrhage and 58 (15.1%) undetermined stroke type. The median National Institutes of Health Stroke Scale on presentation was 17 [interquartile range (IQR) 9–25]. Haemorrhagic strokes compared with ischaemic strokes were more severe, 20 (IQR 12–26) vs. 13 (IQR 7–22) (*p* < 0.001), and occurred in a younger population, mean age 52.3 (SD 12.0) vs. 61.6 (SD 13.8) (*p* < 0.001), with a lower level of educational attainment of 28.2 vs. 40.7% (*p* = 0.04). The median time from stroke onset to arrival at the principal referral hospital was 25 hours (IQR 6–73). Half of the patients (50.4%) sought care at another health provider prior to arrival. One hundred fifty-one patients died in the hospital (39.5%). Forty-three deaths occurred within 48 hours of arriving at the hospital, with median time to death of 4 days (IQR 0–7 days). Of the patients, 49.6% had ≥1 complication, 98 (25.5%) pneumonia and 33 (8.6%) urinary tract infection. Male gender (OR 3.33, 1.65–6.75), pneumonia (OR 3.75, 1.82–7.76), subarachnoid haemorrhage (OR 43.1, 6.70–277.4) and undetermined stroke types (OR 6.35, 2.17–18.60) were associated with higher risk of in-hospital death.

**Discussion:** We observed severe strokes occurring in a young population with high in-hospital mortality. Further work to deliver evidence-based stroke care is essential to reduce stroke mortality in Sierra Leone.

## Introduction

Stroke is the second leading cause of adult death in Sub-Saharan Africa (SSA) (1). Globally, 90% of stroke burden is attributable to modifiable risk factors (2); however, these risk factors vary greatly by region, age and ethnicity (3). Local risk and stroke outcome data are essential to inform the development of stroke services. Stroke studies in SSA are limited in number and design and lack access to imaging (4). The basic understanding of who is suffering from stroke, the outcomes after stroke and the quality of care they receive is limited (5).

The current evidence suggests that stroke occurs at a younger age in SSA compared with high-income regions (5). The mean age of stroke in SSA in the INTERSTROKE case–control study was 57.7, compared with 66.0 in high-income countries (HICs) (3). A review of hospital-based studies in SSA calculated a pooled mean age of stroke of 55 years (6), and the SIREN case–control study of 2,118 case–control pairs in Ghana and Nigeria found a mean age of stroke of 59 years (7). Stroke type reportedly differs in SSA, with higher proportions of haemorrhagic strokes reported; SIREN reported 32% of strokes as haemorrhagic (7); and in stroke patients aged under 50 years, haemorrhagic stroke represented 52.5% of all stroke types (8). A 10-year retrospective hospital-based case series in Nigeria reported 45% of strokes as intracerebral haemorrhage (9), whilst a retrospective hospital-based study in Conakry, Guinea, found 25.2% of strokes were haemorrhagic (10). Hospital outcomes vary widely dependent on country; a recent systematic review estimated a pooled 1-month case fatality of 24.1%, with individual study case fatality varying from 6.6 to 57.6% (11). A systematic review on stroke care in SSA from 2017 found publications that reported stroke care provision from only 14 out of 54 SSA countries (12). Major challenges described are low levels of awareness of stroke warning signs (13); lack of pre-hospital systems; limited number of stroke units, trained personnel and rehabilitation services; and cost of care.

Stroke registers have driven quality improvement in stroke care in many HICs (14), evaluated major health system change for stroke (15) and monitored the uptake of evidence-based care (16). A World Health Organisation (WHO)-led study of stroke registers in low-to-middle-income countries (LMICs) recommended their use to enhance care, prevention and rehabilitation of stroke (17). The Stroke in Sierra Leone (SISLE) programme uses a register approach to improve the uptake of evidence-based care for stroke. We describe the register methodology, stroke types, socio-demographics, hospital outcomes and quality of care indicators and report explanatory models of in-hospital death.

## Setting

The SISLE stroke register is a prospective hospital-based stroke register, based at the principal adult referral hospital in Freetown, Western Area, Sierra Leone. The population of Western Area is 1,500,234, as of 2015 (18). It is the largest hospital in Freetown, a 280-bed facility, with 125 medical beds divided by gender, and a six-bed intensive care unit. The intensive care unit offers continuous cardiac monitoring and a nurse-to-bed ratio of 1:2; mechanical ventilation is rarely available. Medical wards have a nurse-to-bed ratio of 1:4. The hospital is the principal referral hospital for the country, receiving patients from across the country as well as receiving patients directly from the surrounding community of Freetown. There is no formal multidisciplinary team working, and the nursing staff have not received stroke-specific training. There is no stroke unit or practising stroke specialist physician. There are only four trained physiotherapists in the country (19), no speech and language therapist and no occupational therapist. There is no functional CT scan or MRI in the government health system; the CT scans in our study were performed at a private facility and paid for by the project. At the hospital, all formal charges for services, diagnostics and medications need to be paid before the patient can access services. To reduce the cost barrier to access and provide a more representative picture of stroke in Sierra Leone, all investigations in our study were funded by the research funder. All treatment costs, including physiotherapy, were paid for by the patient.

## Methods

The prospective observational hospital-based stroke register design was based on the South London Stroke Register (20) and standard international stroke register methods (17). The register recruited all people with stroke aged 18 years and over presenting at the hospital from May 1, 2019, until April 30, 2020. Stroke was defined according to the WHO definition (21). The register recruited all first-in-a-lifetime strokes and subsequent strokes. All stroke subtypes were included: ischaemic (iCD63); intracerebral haemorrhage (ICD61); subarachnoid haemorrhages (SAHs) (ICD60); and unspecified stroke types (ICD62) (22). Classification of pathological stroke subtype was performed by AR, an experienced stroke physician, with reference to the case history, investigation results and imaging. Cases were classified by the Oxfordshire Community Stroke Project (OCSP) classification (23) and the Trial of Org 10,172 in Acute Stroke Treatment (TOAST) classification (24). Classification was based on results from brain imaging within 30 days of stroke onset (by either CT or MRI scanning). No patients underwent autopsy. Cases without a cause identified with the investigations performed were described as undetermined.

A single 12-lead ECG was performed for all patients on admission. Carotid Doppler, prolonged ECG monitoring and echocardiography were not routinely available (carotid Doppler was performed on three patients, and two patients received echocardiography in our study, performed at a private hospital). Clinicians received formal training on National Institutes of Health Stroke Scale (NIHSS) (25) and performed scoring under supervision of co-investigators until they become proficient. All clinical research staff were trained on the use of the Barthel index (BI) and modified Rankin Scale (mRS). Due to limited access to primary healthcare, for many participants, the stroke admission was their first encounter with the formal healthcare system, and they presented with underlying undiagnosed risk factors. Therefore, we used risk factor definitions (see Supplementary Material) in line with previous international and regional stroke studies (7, 26).

A patient-and-family interview on admission was performed by the study clinician including the NIHSS. The BI and the mRS pre-stroke (measured as the day before onset of stroke symptoms) were collected by observation or caregiver interview at 7 days post admission. At discharge, care processes, complications and final outcome were completed from the patient's clinical record.

All data were collected on standardised paper Case Report Forms. Double data entry was performed, and all data were uploaded onto REDCap™ (27). Statistical analysis was performed in STATA v16, StataCorp™ (28). Continuous variables, with normal distribution, were reported as means and standard deviations (SDs). Ordinal or non-normal variables were reported as medians and interquartile range (IQR). Pearson's chi-squared test was used to examine associations between categorical variables. Unpaired *t*-tests were performed on continuous variables. The Mann–Whitney *U*-test was performed on non-normal/skewed data. A full variable list is provided in the Supplementary Material, and a review of the data demonstrated low levels of missing data.

In the multivariable analysis, we examined the association between gender, educational attainment, ethnicity, complications and hospital mortality. We controlled for age, stroke severity (NIHSS), premorbid status (mRS) and comorbidities. Variables were selected based on known predictors of stroke mortality from regional (10, 11) and international stroke studies (29). Logistic regression was performed, and we report odds ratios (ORs) and 95% CIs. The regression was repeated after classifying and excluding early deaths within 48 hours of arrival.

The study received ethical approval from King's College London (HR-18/19-8467) and approval from the Sierra Leone Ethical and Scientific Review Committee on December 18, 2018. Written consent was sought from all patients. For those unable to give consent, informed consent was sought from the next of kin. A stroke survivors' group was formed alongside the study (30). The stroke survivors' group helped develop participant information leaflets and recruitment documents. The stroke survivors' group will support the dissemination of the research findings to patients and the public in Sierra Leone in an accessible and interpretable manner.

## Results

**Figure 1:**
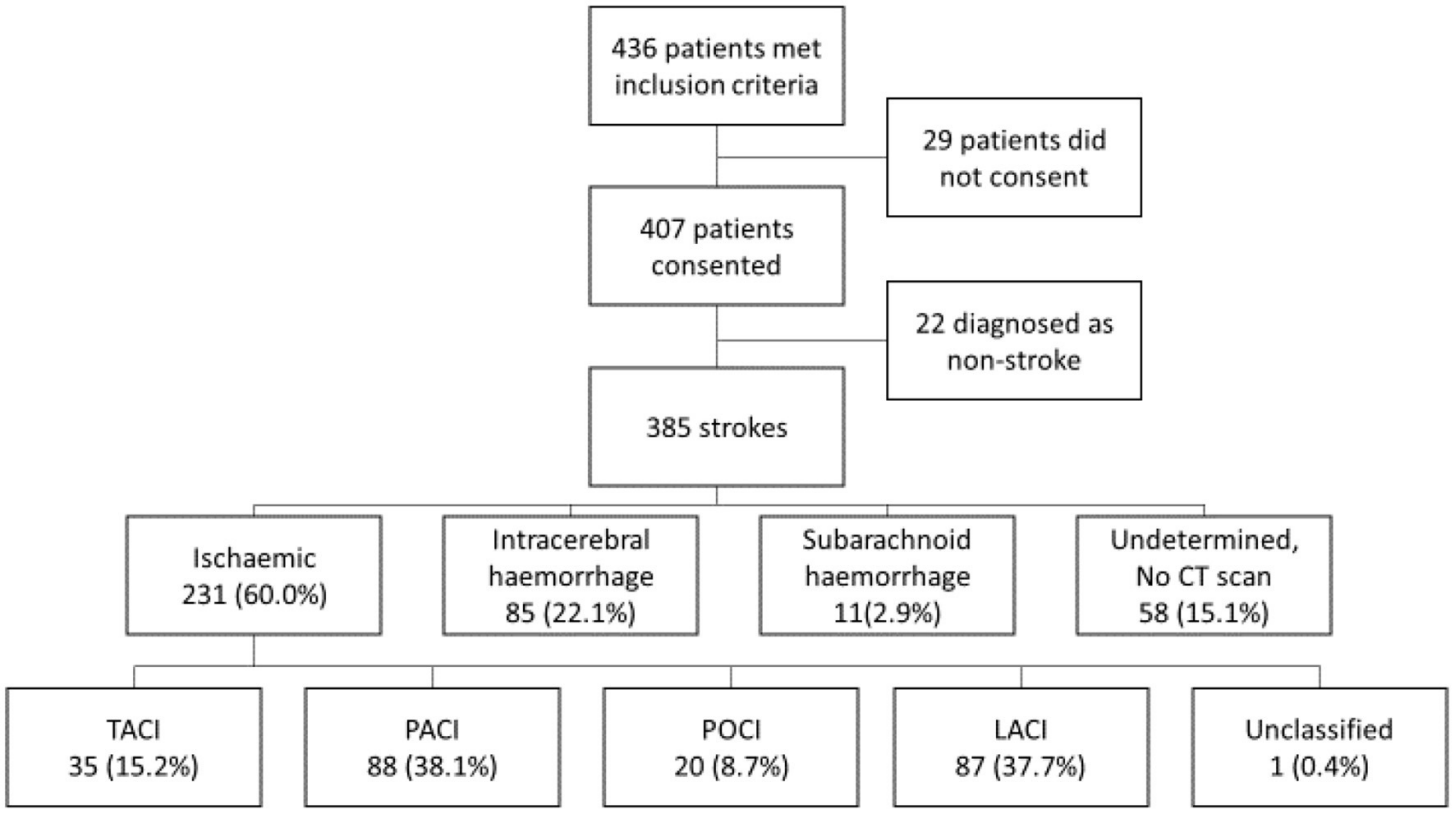
Flowchart of stroke types and Oxford Community Stroke Project (OCSP) classification. TACI, total anterior circulation infarction; PACI, partial anterior circulation infarction; POCI, posterior circulation infarction; LACI, lacunar infarction.

Over the 12-month period, 436 patients met the study inclusion criteria. Twenty-nine patients declined to participate or died before consent could be obtained. Four hundred seven patients consented to the register. After further diagnostic investigation, 22 were subsequently classified as stroke mimics, leaving 385 strokes in the analysis. Of them, 315 (81.8%) were first-in-a-lifetime stroke. The mean age of stroke was 59.2 (SD 13.8), and 187 (48.6%) were male (Table 1). CT scans were performed for 327 (84.9%) patients, and stroke types are detailed in Figure 1.

**Table 1:** Descriptive statistics of stroke by pathological type.

	**All strokes**	**Ischaemic**	**Intracerebral haemorrhage**	**Subarachnoid haemorrhage**	**Undetermined (no CT scan)**	**Missing**
N	385	231 (60.0)	85 (22.1)	11 (2.9)	58 (15.1)	0
First in lifetime stroke	315 (81.8)	188 (81.4)	73 (85.9)	9 (81.8)	45 (77.6)	4
Male	187 (48.6)	116 (50.2)	45 (52.9)	0	26 (44.8)	0
Age mean (SD)	59.2 (13.8)	61.6 (13.8)	52.3 (12.0)	50.2 (12.2)	61.1 (13.2)	4
Ethnicity	378					7
Fullah	22 (5.8)	12 (5.2)	5 (5.9)	1 (9.1)	4 (6.9)	
Krio	52 (13.8)	37 (16.0)	6 (7.1)	1 (9.1)	8 (13.8)	
Limba	50 (13.2)	26 (11.3)	9 (10.6)	2 (18.2)	13 (22.4)	
Mende	61 (16.1)	33 (14.3)	16 (18.8)	2 (18.2)	10 (17.2)	
Temne	131 (34.7)	82 (35.5)	35 (41.2)	2 (18.2)	12 (20.7)	
Other	62 (16.4)	38 (16.5)	12 (14.1)	3 (27.3)	9 (15.5)	
Hypertension	320 (83.1)	188 (81.0)	73 (85.9)	9 (81.8)	50 (86.2)	0
Diabetes	62 (16.1)	47 (20.3)	6 (7.1)	1 (9.1)	8 (13.8)	51
Dyslipidaemia	135 (35.1)	79 (34.2)	36 (42.4)	4 (36.4)	16 (27.6)	5
Atrial Fibrillation	18 (4.7)	11 (4.8)	2 (2.4)	0	5 (8.6)	34
Current smoker	54 (14.0)	38 (16.5)	9 (10.6)	0	7 (12.1)	6
Alcohol use	98 (26.5)	68 (18.4)	17 (4.6)	0	13 (3.5)	15
Waist-to-hip ratio	314					71
Normal	116	75 (32.5)	24 (28.2)	3 (27.3)	14 (24.1)	
Moderate	101	59 (25.5)	25 (29.4)	4 (36.4)	13 (22.4)	
High	97	63 (27.3)	19 (22.4)	3 (27.3)	12 (20.7)	
Higher education level	142 (36.9)	94 (41.6)	24 (28.9)	2 (18.2)	22 (39.9)	9
Pre-stroke Barthel Index Median and IQR	100 (100–100)	100 (100–100)	100 (100–100)	100 (100–100)	100 (100–100)	2
Pre-stroke modified Rankin Score Median and IQR	0 (0–0)	0 (0–0)	0 (0–0)	0 (0–0)	0 (0–0)	2
National Institute of Health Stroke Severity Scale (median and IQR)	17 (9–25)	13 (7–22)	20 (12–26)	12 (4–25)	28 (24–31)	2
Mild stroke ≤ 6	62 (16.1)	46 (12.0)	9 (2.4)	4 (1.0)	3 (0.8)	
Moderate stroke 7–16	126 (32.7)	95 (24.8)	24 (6.3)	3 (0.8)	4 (1.0)	
Severe stroke >16	195 (50.6)	88 (23.0)	52 (13.6)	4 (1.0)	51 (13.3)	
In hospital Death	151 (39.2)	59 (25.5)	37 (43.5)	6 (54.5)	49 (84.5)	0
Post stroke modified Rankin Score (median and IQR)	5 (IQR 4–6)	4 (4–6)	5 (4–6)	6(4–6)	6 (6–6)	7
Post stroke Barthel Index (median and IQR)	15 (0–45)	25 (0–45)	0 (0–0)	0 (0–0)	0 (0–0)	
Complication ≥ 1	191 (49.6)	100 (43.3)	39 (45.9)	7 (63.6)	45 (83.3)	
Pneumonia	98 (25.5)	50 (21.6)	19 (22.4)	1 (9.1)	28 (48.3)	
Urinary tract infection	33 (8.6)	23 (10.1)	8 (9.4)	1 (9.1)	1 (1.7)	
Seizures	29 (7.5)	14 (6.1)	4 (4.7)	1 (9.1)	10 (17.2)	
Pressure sores	12 (3.1)	8 (3.5)	1 (1.2)	1 (9.1)	2 (3.4)	
Deep vein thrombosis	5 (1.3)	2 (0.9)	2 (2.4)	0	1 (1.7)	

*Data are count (%) unless otherwise indicated*.

Of the patients, 168 (45.8%) were reported to be the main breadwinner for their family; 135 (36.4%) were in full-time employment, 18 (4.9%) were in part-time and 133 (35.9%) were retired. Of the patients, 142 (36.9%) had a higher educational level.

**Figure 2:**
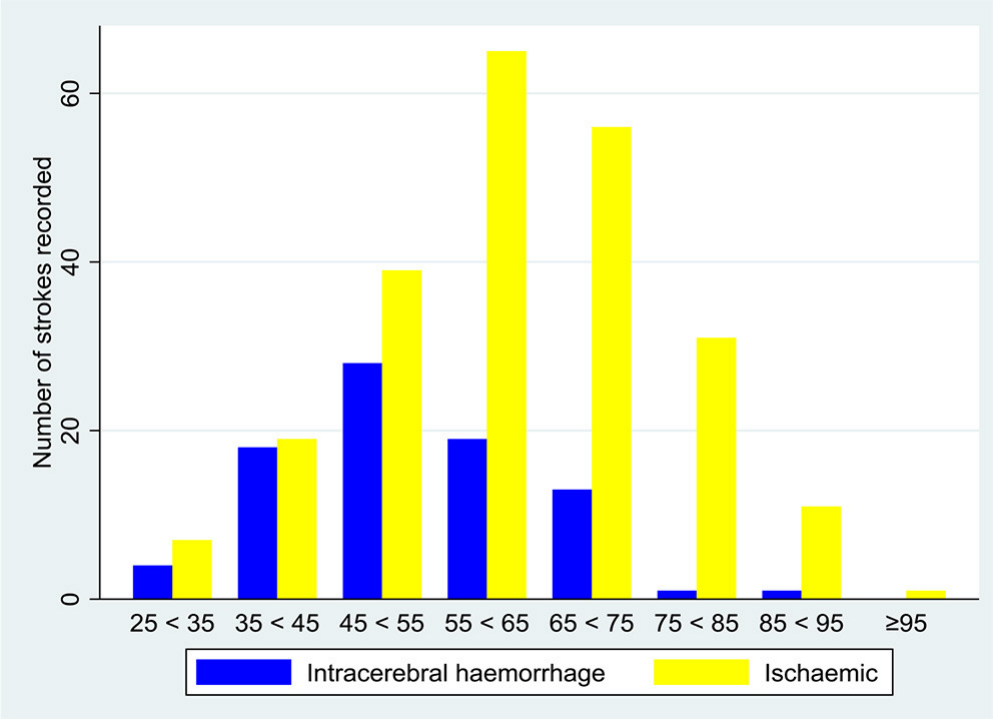
Number of stroke cases by subtype and 10-year age groups.

Intracerebral haemorrhages occurred in younger patients: 52.3 (12.0) years compared with 61.6 (13.8) years for ischaemic strokes (*p* < 0.001) (Figure 2). Intracerebral haemorrhages were more severe than ischaemic strokes, with NIHSS at admission 20 (12–26) and 13 (7–22), respectively (*p* < 0.001). In a univariate analysis, haemorrhagic strokes had a higher in-hospital mortality 43.5% compared with 25.5% in ischaemic strokes (*p* = 0.002) (Supplementary Material).

The median time from stroke onset to arrival at the principal adult referral hospital was 25 hours (IQR 0–68). Half of the patients (50.4%) sought care at another health provider beforehand (Table 2). Time from stroke onset to arrival if the patient came directly to Connaught Hospital was 12.5 hours compared with 51 hours if the patient sought care elsewhere before presenting at Connaught (*p* < 0.0001). The majority of the patients who sought care prior to arriving at Connaught visited a referral hospital (65.5%). There were no significant associations between time to arrival and transport type used.

**Table 2:** Care provision.

**Pre-Hospital care**	
Sought care before Connaught Hospital	194 (50.4%)
**Care provider**	
Referral hospital	127 (65.5%)
Peripheral health unit	32 (16.5%)
Healthcare worker in community	21 (10.8%)
Pharmacist	4 (2.1%)
Traditional healer	4 (2.1%)
Other	6 (3.1%)
**Mode of transport of arrival**	
Keke (3 wheel rickshaw)	98 (25.5%)
Taxi	89 (23.1%)
Ambulance	78 (20.3%)
Own vehicle	72 (18.7%)
Other	8 (2.1%)
Motor bike	4 (1.0%)
Walking	3 (0.8%)
**Ongoing care after Emergency Department**	
Medical Ward Admission	302/385 (78.4%)
ICU admission	14/385 (3.6%)
Died in Emergency department	56/385 (14.5%)
Outpatient	9/385 (2.3%)
**Quality of care indicators**	
GCS recorded on admission	251/385 (65.2%)
Antiplatelets prescribed for ischaemic stroke	144/231 (62.3%)
Statin prescribed if dyslipidaemia	93/139 (66.9%)
Referred to physiotherapy	179 (76.8%)
Physiotherapy received in hospital	58/385 (15.1%)
Documented Dysphagia Assessment	3/385 (0.8%)
Documented risk factor advice for those surviving to discharge	33 (14.1%)

Of the patients, 151 (39.5%) died in the hospital: 143 of those who died had data on time of death and time of arrival; 43 deaths (30.1%) occurred within 48 hours of arriving at the hospital, and the median time to death was 4 days (IQR 0–7 days). The median time to discharge was 9 days (4–15). Those who survived to discharge left the hospital with significant disability, with a median mRS of 4.0 (IQR 4–5) and median BI of 25 (IQR 10–50). Of the patients, 179 (76.8%) surviving to discharge were referred to physiotherapy, and 15.1% of the patients received physiotherapy as an inpatient. The median time from arrival to referral to physiotherapy was 81 hours (IQR 39–123). The median time for patients to receive physiotherapy after referral was 32 hours (IQR 0–88). The median time from arrival to CT scan was 28 hours (IQR 11–56). Of the patients, 49.6% had ≥1 complication, 98 (25.5%) pneumonia and 33 (8.6%) urinary tract infection (Table 1). Of the pneumonias, 43/98 (43.9%) were diagnosed on the same day as the patient arrived.

In a multivariable analysis, male gender was associated with an increased risk of death, OR 3.33 (1.65–6.75) (Table 3). Patients diagnosed with diabetes were at increased risk of death, OR 2.35 (1.02–5.46). Relative to ischaemic stroke, SAH and undetermined stroke types had a higher chance of death, OR 43.1 (6.70–277.4) and OR 6.35 (2.17–18.60), respectively. Pneumonia was the only post-stroke complication associated with increased risk of in-hospital death, OR 3.75 (1.82–7.76).

**Table 3:** Multivariable analysis of odds ratios for in-hospital death.

**Variable**	**Multivariable analysis OR**	**95% Cis**	***P*-value**
Age	1.00	0.98–1.02	0.967
Male	3.33	1.65–6.75	0.001
**Ethnicity**			
Temne	Ref		
Fullah	0.11	0.03–0.49	0.003
Krio	0.78	0.28–2.21	0.64
Limba	0.63	0.24–1.62	0.34
Mende	0.86	0.33–2.22	0.76
Other	0.70	0.27–1.80	0.51
Higher educational level	0.81	0.39–1.66	0.56
Hypertension	1.46	0.62–3.42	0.38
Diabetes	2.35	1.02–5.46	0.046
Atrial fibrillation	0.49	0.11–2.17	0.35
National Institutes of Health Stroke Scale	1.22	1.16–1.28	<0.001
**Stroke type**			
Ischaemic	Ref		
Intracerebral haemorrhage	1.61	0.74–3.48	0.23
Subarachnoid haemorrhage	43.1	6.70–277.4	<0.001
Undetermined	6.35	2.17–18.60	<0.001
Pneumonia	3.75	1.82–7.76	<0.001
Seizures	0.94	0.27–3.22	0.92
Urinary tract infection	0.0.51	0.17–1.54	0.23
Pressure sores	1.76	0.40–7.78	0.46

## Discussion

This study aimed to describe the basic demographics, stroke types, care provision and hospital outcomes for stroke in Freetown, Sierra Leone. We report severe strokes occurring at a young age in previously fully independent individuals. Our reported in-hospital mortality of 39.5% is at the higher end of the 14.4–50% range reported from hospital-based studies in SSA (11) and significantly higher than that of HIC registers (31). High stroke severity, stroke types, high rates of pneumonia and inadequate care are all contributing to in-hospital mortality.

Our stroke patients were significantly younger than seen in stroke registers in HICs and younger than reported in an international stroke register study in low-to-middle-income countries (LMICs). Strikingly, the majority of our patients were fully functionally independent before their stroke, and 45.8% were the main breadwinner for their household. Our patients suffered severe strokes, higher than other West African hospital-based stroke registers (12, 22) and significantly higher than HIC stroke registers (32). Haemorrhagic strokes accounted for 26.0% of strokes with known stroke type, similar to international studies and studies from neighbouring Guinea (12) but lower than the 32% reported by SIREN (9). Haemorrhagic strokes were associated with increased mortality in a univariable but not multivariable analysis. Compared with ischaemic strokes, the higher risk mortality for SAH may be explained by patients presenting with headache and meningism but limited neurological signs. These patients also presented with relatively low NIHSS scores, followed by rapid deterioration, with an absence of neurosurgical intervention available to prevent deterioration. Undetermined stroke type, OR 6.35 (2.17–18.60), is explainable, as these are patients whose clinical condition precluded transfer to CT scan, which is located at another hospital and requires ambulance transfer.

Patients arrived late, and many patients presented with aspiration pneumonia on arrival. Almost half, 49.6%, of all patients had a stroke-related complication. Pneumonia was the most prevalent complication, reported in 25.5% of the patients, double the 12.3% rate reported in a recent systematic review of 139,432 acute strokes (33). Dysphagia is more likely to occur in severe strokes, and the mean NIHSS in the systematic review was 8.2, compared with our 17.2. Importantly, <1% of stroke patients in our study had a documented dysphagia assessment. The low rate of dysphagia assessment should be a key focus for a quality-improvement programme. Multi-centre trials have shown that formal dysphagia screens prevent stroke-associated pneumonia even after adjusting for stroke severity (34), and delays in dysphagia assessment lead to higher rates of stroke-associated pneumonia (35).

Only a proportion of the patients are receiving the care needed to improve stroke outcomes, and care falls short of the essential stroke services standard of the World Stroke Organization's Roadmap for delivering quality stroke care (36). Over a third of ischaemic strokes were not prescribed aspirin as acute therapy or for secondary prevention. One third of the patients with dyslipidaemia were not prescribed statin therapy. Whilst 77.2% of those who survived to discharge were referred to physiotherapy, only 15.1% of the patients received physiotherapy as an inpatient. The median time to receive therapy was 81 hours, with patients therefore not receiving the benefits of early mobilisation. The high in-hospital mortality, high rate of complications and the relatively low uptake of evidence-based stroke care guidelines underline the case to invest in improvements in stroke services in Sierra Leone. A priority step toward improving stroke services would be the introduction of stroke unit-based care (36).

The introduction of stroke unit-based care has been the most effective method to improve mortality and morbidity in HICs (37). However, there are significant barriers to developing stroke unit-based care, in low-income countries (LICs) such as Sierra Leone (38). For example, how can multi-disciplinary stroke care be introduced if there are limited numbers of physiotherapists and an absence of other allied health professionals. Understanding the essential elements of stroke care to focus on, which can then be delivered by a non-specialist nurse workforce, may prove a promising approach (39). Despite these challenges, a recent before-and-after study of 679 patients reports a mortality reduction from 22.3 to 7.2% after the introduction of minimal setting stroke care in neighbouring Conakry, Guinea (10). However, this study has been criticised for survivor bias. In our setting, in-hospital interventions will need to be complimented with pre-hospital interventions. Improving referral pathways with peripheral hospitals to encourage earlier referral, community education programmes that encourage patients to present earlier and prevent pre-hospital aspiration pneumonia, combined with initiatives to reduce the cost barrier to access, are all candidate interventions.

Our study further builds on the evidence base for stroke in Sierra Leone (40, 41). The study used international and locally appropriate risk factor definitions, standardised assessments by well-trained staff and achieved high rates of CT scanning and high rates of data completeness. Our study is limited by its hospital-based design, which prevents extrapolations of findings to the population level. Hospital-based studies, in contrast to population-based studies, consistently report lower stroke incidence due to underdetection of strokes (5, 6). The cost of care in Sierra Leone likely biases recruitment toward higher socio-economic groups, those with more severe strokes and those living in closer proximity to the hospital. We attempted to mitigate this through paying for all stroke-related investigations and admission to the hospital; however, all treatment costs, including physiotherapy, were borne by the patient. The absence of more detailed investigations and extended ECG or cardiac monitoring, echocardiography and carotid Doppler mean that our study had limited ability to define aetiology of stroke. The absence of CT scanning at our facility meant that the sickest patients who fulfilled our inclusion criteria were unable to be transferred to receive CT imaging. Further research from the SISLE register will describe long-term outcomes after stroke and examine interventions to improve the quality of stroke care.

## Conclusion

We report severe strokes occurring in young, previously fully able individuals and high hospital mortality at 39.5%. High stroke severity, stroke type mix, high rates of pneumonia and inadequate care are all contributing to high in-hospital mortality. Improvements in quality of care, alongside interventions to encourage patients to attend the hospital earlier, and to prevent aspiration pneumonia both before and in the hospital, are essential to reduce stroke mortality in Sierra Leone.

## Data Availability Statement

The raw data for this study contain both personally identifiable and confidential clinical data. Requests for data access for academic use should be made to the SISLE team where data will be made available subject to academic review and acceptance of a data-sharing agreement. https://www.kcl.ac.uk/research/stroke.

## Ethics Statement

This study involved human participants and received ethical approval from King's College London (HR-18/19-8467) and approval from the Sierra Leone Ethical and Scientific Review Committee on 18th December 2018. The participants provided their written informed consent to participate in this study.

## Author Contributions

DY is first author and wrote the first draft. DY, GD, JF-R, PL, AL, IM, AR, YW, CWa, CWo, DL, and CMS designed the research. DY, GD, EB, IJ, AS, CS, MT, DL, and CMS conducted the research. DY conducted the statistical analysis in consultation with IM, JF-R, HW, and YW. All authors contributed to the article and approved the submitted version.

## Funding

This research was funded by the National Institute for Health Research (NIHR) (GHR: 17:63:66) using UK aid from the UK Government to support global health research. The views expressed in this publication are those of the authors and not necessarily those of the NIHR or the UK Department of Health and Social Care. CWo, JF-R, and YW received funding from the National Institute for Health Research (NIHR) Biomedical Research Centre (BRC), Guy's and St Thomas' NHS Foundation Trust and King's College London, London, United Kingdom, and the NIHR Applied Research Collaboration (ARC) South London, London, United Kingdom.

## Conflict of Interest

The authors declare that the research was conducted in the absence of any commercial or financial relationships that could be construed as a potential conflict of interest.

## Publisher's Note

All claims expressed in this article are solely those of the authors and do not necessarily represent those of their affiliated organizations, or those of the publisher, the editors and the reviewers. Any product that may be evaluated in this article, or claim that may be made by its manufacturer, is not guaranteed or endorsed by the publisher.
