# Supplementary material for: A Prospective Stroke Register in Sierra Leone: Demographics, Stroke Type, Stroke Care and Hospital Outcomes
Source: Front Neurol. 2021 Sep 7;12:712060. doi: 10.3389/fneur.2021.712060 (PMC8453059; doi:10.3389/fneur.2021.712060)
Supplement: Supplementary file 1 [file Data_Sheet_1.docx]

**Supplementary Material**

|  | Ischaemic  n=231 | Intracerebral haemorrhage  n=85 | P value |
| --- | --- | --- | --- |
| Male (%) | 116 (50.2%) | 45 (52.9%) | 0.667 |
| Age, mean (SD) | 61.6  (13.8) | 52.3  (12.0) | <0.001 |
| Ethnicity  Fullah  Krio  Limba  Mende  Temne  Other |  |  | 0.40 |
|  | 12 (5.2%) | 5 (5.9%) |  |
|  | 37 (16.0%) | 6 (7.1%) |  |
|  | 26 (11.3%) | 9 (10.5%) |  |
|  | 33 (14.3%) | 16 (18.8%) |  |
|  | 82 (35.5%) | 35 (41.2%) |  |
|  | 79 (34.2%) | 28 (32.9%) |  |
| Pre stroke modified Rankin Score <3 | 209 (90.5%) | 81 (95.3%) | 0.087 |
| Pre stroke Barthel Index median (IQR) | 100 (100-100) | 100  (100-100) | <0.001* |
| Prior stroke | 41 (17.7%) | 10 (11.8%) | 0.388 |
| Hypertension | 188 (81.0%) | 73 (85.9%) | 0.35 |
| Diabetes | 47 (20.3%) | 6 (7.1%) | 0.006 |
| Dyslipidaemia | 79 (34.2%) | 36 (42.4%) | 0.259 |
| Atrial Fib | 11 (4.8%) | 2 (2.4%) | 0.339 |
| HIV | 7 (3.0%) | 5 (5.9%) | 0.674 |
| Waist Hip ratio  Normal  Moderate increase  High increase |  |  | 0.57 |
|  | 75 (32.5%) | 24 (28.2%) |  |
|  | 59 (25.5%) | 25 (29.4%) |  |
|  | 63 (27.3%) | 19 (22.4%) |  |
| Education level high | 94 (40.7%) | 24 (28.2%) |  |
| Alcohol use | 68 (29.4%) | 17 (20.0%) | 0.114 |
| Current smoker | 38 (16.5%) | 9 (10.6%) | 0.205 |
| Time from stroke onset to arrival in hours, median (IQR) | 31 (IQR 11-84) | 15 (IQR 3-37) | <0.001* |
| Admission NIHSS, median (IQR) | 13  (IQR 7-22) | 20  (IQR 12-26) | <0.001 |
| In Hospital Death | 59 (25.5%) | 37 (43.5%) | 0.002 |
| Post stroke modified Rankin Score high  (3-6) | 200 (90.5%) | 80 (94.1%) | 0.074 |
| Post stroke Barthel Index median (IQR) | 0  (0-0) | 0 (0-0) | 0.006* |
| Complication ≥1  Pneumonia  Urinary tract infection  Seizures  Pressure sores  Deep vein thrombosis | 100 (43.3%) | 39 (45.9%) | 0.68 |
|  | 50 (21.6%) | 19 (22.4%) | 0.85 |
|  | 23 (10.0%) | 8 (9.4%) | 0.89 |
|  | 14 (6.1%) | 4 (4.7%) | 0.65 |
|  | 8 (3.5%) | 1 (1.2%) | 0.28 |
|  | 2 (0.8%) | 2 (2.4%) | 0.29 |

*Supplementary material Table 1: Factors associated with ischaemic stroke and intracerebral haemorrhage*

**Mann Whitney U Test.*

**Statistical Analysis Plan**

**Statistical analysis plan**

Time Period for analysis: 01/05/2019 – 31/04/2020. Justification: The SISLE stroke register began with a one month pilot period in April 2018. The pilot period ensured that case ascertainment methods were reinforced, raised awareness of the register amongst clinicians and tested recruitment processes and pathways of access to clinical investigations.

The overall aim of this analysis plan is to provide a description of the natural history of stroke in Freetown, Sierra Leone. We will describe the demographics of stroke by stroke subtype and evaluate stroke in hospital mortality.

**Objectives and hypothesis:**

1. To describe the basic demographics, risk factors and prevalence of stroke subtypes, in a hospital-based register in Sierra Leone. *Hypothesis: There is a difference in risk factors and basic demographics between haemorrhagic and ischaemic strokes in Sierra Leone*
2. To analyse which variables are related to in hospital stroke mortality. *Hypothesis: Certain variables will be associated with in hospital mortality.*

**Descriptive characteristics of cohort**

Age in years will be categorised into mid-decade ten year age bands as standard for stroke incidence studies^17^. Sex will remain as categorical male / female. Hypertension, diabetes, dyslipidaemia, hip to waist ratio will be categorised as present or absent as per the definitions above. Education level ordinal variables 0-6 will be transformed to categorical variable 0-2 and 3-6, cut off will be finishing secondary school. Across stroke studies the NIHSS is used both as a continuous variables as well as categorical^18^. There are a variety of different cut offs for NIHSS across publications for how to classify mild moderate and severe stroke. We used mild 0-6, moderate 7-16 and severe 17-40 as our cut-offs, based on global stroke comparison studies^19^. T-test or Mann Whitney test will be used to compare groups, dependent on distribution.

**Variable Definitions**

- Ethnicity was self-reported and categorised as per Sierra Leone census groupings(17). For analysis we included the five most frequent ethnic categories in our dataset and coded the remainder as “other”.
- Hypertension was defined as: serial blood pressure readings ≥140/90mmHg from 72 hours after stroke or; patient reported history of hypertension or; history of antihypertensive use or continuing use of antihypertensives 72 hours post stroke.
- Type two diabetes mellitus was defined as: previous documented history of diabetes or patient reported history of diabetes; previous prescription of diabetic medication; or a HbA1C >6.5% on admission.
- Dyslipidaemia was defined as; previous history of dyslipidaemia or; previous prescription of lipid controlling drugs; or any of a fasting total cholesterol concentration of ≥5·2 mmol/L, HDL cholesterol ≤1·03 mmol/L, triglyceride ≥1·7 mmol/L, or LDL cholesterol ≥3·4 mmol/L on admission.
- Participants were categorised as current smokers, ex-smokers or never smoked. Smoking exposure was recorded in pack years.
- Participants were categorised as current alcohol drinkers, ex drinkers or never drank alcohol and alcohol exposure was calculated as units/week.
- Waist-to-hip ratio was categorised using the INTERSTROKE classifications as low (≤0.91), moderate (0.91-0.96), or high (≥0.97) for males, and low (≤0.86), moderate (0.86- 0.93), or high (≥0.93) for females.
- Highest level of educational attainment was classified as; no school; completion of primary school; completion of Basic Education Certificate Examination (BECE); completion of West African Senior school certificate examination (WASCE); graduate degree; and Masters degree or higher. A binary variable for educational attainment was created, with the cut-off completion of high school (WASCE).
- Referral hospital was defined as tertiary and district government hospitals nationwide, or community health centres in the Western Area.
